# Preserved SARS-CoV-2 Vaccine Cell-Mediated Immunogenicity in Patients With Inflammatory Bowel Disease on Immune-Modulating Therapies

**DOI:** 10.14309/ctg.0000000000000484

**Published:** 2022-03-28

**Authors:** Brigid S. Boland, Benjamin Goodwin, Zeli Zhang, Nathaniel Bloom, Yu Kato, Jennifer Neill, Helen Le, Tiffani Tysl, Angelina E. Collins, Parambir S. Dulai, Siddharth Singh, Nghia H. Nguyen, Alba Grifoni, Alessandro Sette, Daniela Weiskopf, John T. Chang, Jennifer M. Dan

**Affiliations:** 1 Division of Gastroenterology, University of California, San Diego, La Jolla, California, USA;; 2 Center for Infectious Disease and Vaccine Research, La Jolla Institute for Immunology, La Jolla, California, USA;; 3 Department of Medicine, Division of Infectious Diseases and Global Public Health, University of California, San Diego, La Jolla, California, USA.

## Abstract

Immune-modulating medications for inflammatory bowel diseases (IBDs) have been associated with suboptimal vaccine responses. There are conflicting data with SARS-CoV-2 vaccination. We therefore assessed SARS-CoV-2 vaccine immunogenicity at 2 weeks after second mRNA vaccination in 29 patients with IBD compared with 12 normal healthy donors. We observed reduced humoral immunity in patients with IBD on infliximab. However, we observed no difference in humoral and cell-mediated immunity in patients with IBD on infliximab with a thiopurine or vedolizumab compared with normal healthy donors. This is the first study to demonstrate comparable cell-mediated immunity with SARS-CoV-2 vaccination in patients with IBD treated with different immune-modulating medications.

## INTRODUCTION

Patients with inflammatory bowel diseases (IBDs), specifically Crohn's disease or ulcerative colitis, are frequently treated with immune-modulating therapies to dampen an overactive immune response. Although effective for induction and maintenance of IBD, tumor necrosis factor inhibitors, such as infliximab (IFX), are associated with lower antibody titers to the pneumococcal, influenza, and hepatitis B vaccines (1–3). Studies suggest that gut-specific biologics, such as the integrin antagonist vedolizumab (VDZ), do not result in lower antibody titers to vaccines (4,5). With the SARS-CoV-2 vaccines, concerns emerged over the impact of these immune-modulating therapies, particularly tumor necrosis factor inhibitors in patients with IBD in generating effective immune responses, because the initial efficacy trials did not include individuals on immunomodulating agents.

A large UK study of SARS-CoV-2-infected IBD patients reported significantly lower SARS-CoV-2 seroconversion rates in patients on IFX compared with those on VDZ (6). Two other studies reported that most patients, regardless of immune-modulating therapies, had detectable SARS-CoV-2 spike receptor-binding domain (RBD) immunoglobulin (Ig) G titers after vaccination (7,8). However, none of these studies evaluated neutralizing titers or cell-mediated immunity. The goal of this study was to evaluate SARS-CoV-2 vaccine-induced humoral and cell-mediated immunity in patients with IBD on immune-modulating agents—IFX, IFX with a thiopurine, and VDZ—and normal healthy donors (NHDs).

## METHODS

Protocols were approved by institutional review boards at the University of California, San Diego (IRB# 210093) and La Jolla Institute for Immunology (VD-214). Blood was collected prevaccination and 2 weeks after second vaccination (see Supplemental Methods). Ten of 29 patients with IBD were able to provide prevaccination blood samples. As a comparator, we enrolled 12 NHDs who received either Moderna or Pfizer vaccine. Samples were obtained between March and May 2021 before the emergence of significant variants.

## RESULTS

We enrolled 29 adult patients with IBD, 19 with Crohn's disease, and 10 with ulcerative colitis (Table 1). All patients were on biologics: IFX (n = 9), IFX in combination with azathioprine or 6-mercaptopurine (n = 9), or VDZ (n = 11). All patients received 2 doses of either mRNA-1273 (NIH-Moderna) or BNT 162b2 (Pfizer-BioNTech) SARS-CoV-2 spike mRNA vaccines. Immunologic responses were tested at 2 weeks after second vaccination.

**Table 1.** Baseline demographics

	Patients with IBD (n = 29)	Normal healthy donors (n = 12)
Age, median (IQR)	37 (28–56)	42.5 (28–57)
Sex, n (%)		
Male	14 (48.3)	4 (33.3)
Female	15 (51.7)	8 (66.7)
Race, n (%)		
Asian	4 (13.8)	3 (25)
Black	1 (3.4)	0
Hispanic/Latino	2 (6.9)	0
White	20 (68.9)	7 (58.3)
Other	2 (6.9)	2 (16.7)
Vaccine type, n (%)		
Moderna	21 (72.4)	5 (41.7)
Pfizer	8 (27.6)	7 (58.3)
Disease subtype, n (%)		
Crohn's disease	19 (70.4)	NA
Ulcerative colitis	10 (29.6)	NA
IBD medications, n (%)		
Infliximab	9 (31.0)	NA
Infliximab combination	9 (31.0)	NA
Vedolizumab	11 (37.9)	NA

IBD, inflammatory bowel disease; IQR, interquartile range; n, number; NA, not available.

**Figure 1.**
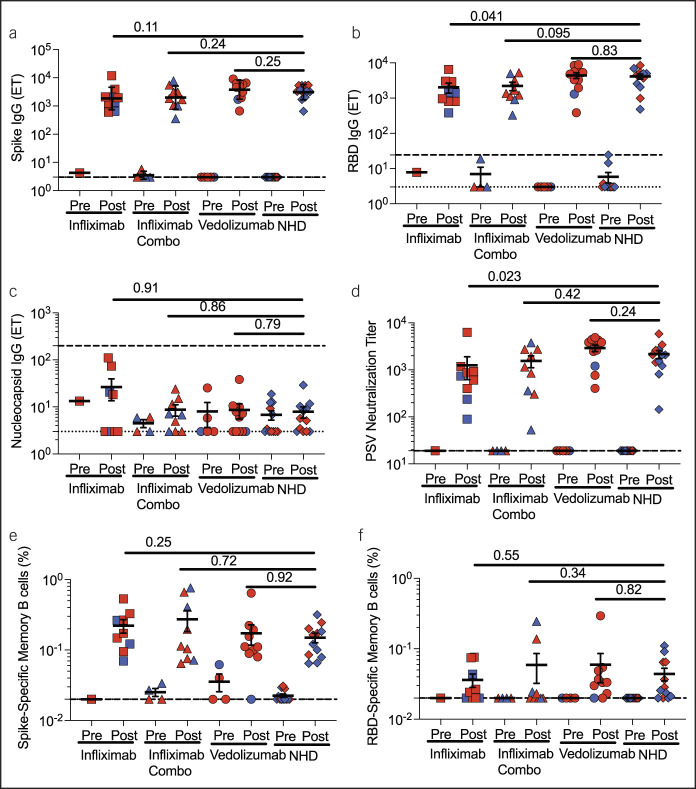
Humoral immune responses to the SARS-CoV-2 vaccine in patients with inflammatory bowel disease (IBD). (**a**) Spike IgG, (**b**) RBD IgG, (**c**) nucleocapsid IgG titers by ELISA, and (**d**) pseudovirus-neutralizing titers for patients with IBD on infliximab (n = 9), infliximab combination therapy (n = 9), and vedolizumab (n = 11) and normal healthy donors (NHDs, n = 12). There were no differences in the frequency of (**e**) spike-specific and (**f**) receptor-binding domain (RBD)-specific memory B cells between patients with IBD and normal healthy donors. Frequencies of postvaccine responses were compared between patients with IBD on their respective biologics and NHDs using the Mann-Whitney test. Red dots indicate recipients of mRNA-1273 (NIH-Moderna); blue dots indicate recipients of the BNT 162b2 (Pfizer-BioNTech) vaccine. Dotted lines represent limit of detection for assay; dashed lines represent limit of sensitivity for assay. ET = endpoint titer.

We first measured SARS-CoV-2 vaccine titers. All patients made comparable SARS-CoV-2 spike IgG (Figure 1a) and spike RBD IgG titers (Figure 1b) at 2 weeks after second vaccination except for IFX monotherapy patients who had significantly lower RBD IgG titers (*P* = 0.041, Figure 1b). All patients had undetectable nucleocapsid IgG titers (Figure 1c), suggesting no one was previously infected with SARS-CoV-2 because the nucleocapsid protein is not encoded by the SARS-CoV-2 vaccine. We next determined whether patients with IBD made comparable pseudovirus-neutralizing titers, which may correlate with protection against SARS-CoV-2 infection (9,10). IFX monotherapy patients had significantly lower neutralizing titers than NHDs (*P* = 0.023, Figure 1d), consistent with lower RBD IgG titers (Figure 1b). Patients with IBD on IFX combination therapy patients or VDZ had comparable titers compared with NHDs (Figure 1d). Finally, we measured the frequency of SARS-CoV-2 spike-specific memory B cells (CD27^+^IgD^−^CD19^+^ cells) and observed comparable frequencies of spike-specific and RBD-specific memory B cells (Figure 1e,f).

**Figure 2.**
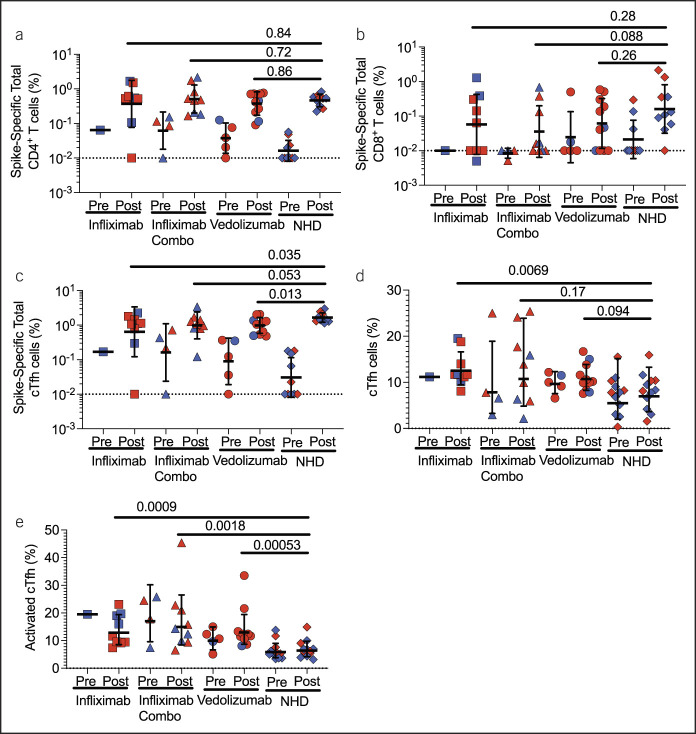
Cell-mediated immune responses to the SARS-CoV-2 vaccine in patients with inflammatory bowel disease (IBD) . Comparable frequencies of SARS-CoV-2 specific total (**a**) CD4^+^ and (**b**) CD8^+^ T cells in patients with IBD and normal healthy donors (NHDs, n = 10). (**c**) Patients with IBD have lower frequencies of SARS-CoV-2 spike-specific cTfh cells than NHDs. (**d**) Patients with IBD on infliximab have a higher frequency of cTfh cells than NHDs. (**e**) Patients with IBD on infliximab, infliximab combination therapy, and vedolizumab had higher frequencies of activated cTfh cells (PD-1^+^ICOS^+^ of CXCR5^+^CD45RA^−^CD4^+^ T cells). The dotted line represents the limit of detection (LOD) at 0.01%. Frequencies of postvaccine responses were compared between patients with IBD on their respective biologics and normal healthy donors using the Mann-Whitney test. Red dots indicate recipients of mRNA-1273 (NIH-Moderna); blue dots indicate recipients of the BNT 162b2 (Pfizer-BioNTech) vaccine. Dotted lines represent LOD for assay.

We next quantified SARS-CoV-2 spike-specific T-cell responses with the activation-induced marker assay using a SARS-CoV-2 spike peptide megapool (11). By coexpression of activation markers OX40 and CD40L, we observed comparable frequencies of SARS-CoV-2 spike-specific CD4^+^ T cells (Figure 2a). By coexpression of activation markers CD69 and 4-1BB, we also observed comparable frequencies of SARS-CoV-2 spike-specific CD8^+^ T cells (Figure 2b).

To determine why IFX monotherapy patients had significantly lower spike RBD IgG and neutralizing titers, we evaluated the frequencies of circulating T follicular helper (cTfh) cells. Tfh cells are CD4^+^ T cells which provide help to B cells to generate antigen-specific IgG responses (12). We first measured SARS-CoV-2 spike-specific cTfh cells (OX40^+^CD40L^+^ of CXCR5^+^CD45RA^−^CD4^+^ T cells) and observed fewer spike-specific cTfh cells in patients with IBD on IFX monotherapy (*P* = 0.035, Figure 2c), IFX combination therapy (*P* = 0.053, Figure 2c), and VDZ (*P* = 0.013, Figure 2c) than NHDs. We then assessed the frequency of cTfh cells (%). Patients on IFX monotherapy had more cTfh cells than NHDs (*P* = 0.0069, Figure 2d). However, patients with IBD on IFX combination therapy and VDZ had comparable cTfh frequencies with NHDs. We finally assessed the frequency of recently activated cTfh cells (PD-1^+^ICOS^+^ of CXCR5^+^CD45RA^−^CD4^+^ T cells). Activated cTfh cells are primed to provide help to B cells (13). Overall, patients with IBD had a higher frequency of activated cTfh cells, regardless of medications, compared with NHDs (Figure 2e).

## DISCUSSION

We report that patients with IBD have preserved spike-specific CD4^+^ and CD8^+^ T cells at 2 weeks after second mRNA vaccination with only reduced RBD and neutralizing titers in patients with IBD on IFX monotherapy. The patients with IBD on IFX monotherapy had a younger age at diagnosis and longer disease duration compared with the IFX combination group, suggesting a more aggressive disease phenotype which may have influenced antibody titers. Furthermore, it is unclear why IFX monotherapy patients had comparable spike IgG titers, but this may be due to pre-existing endemic coronavirus spike-specific memory B cells because there was no difference in the frequency of spike-specific memory B cells (14). Although IFX monotherapy patients had comparable RBD-specific memory B cells, most were at the limit of detection for the assay.

Notably, we observed higher frequencies of activated cTfh cells in all patients with IBD compared with NHDs. This has been observed in other autoimmune diseases in which Tfh cells play a pathogenic role. Activated cTfh cells and tissue-resident Tfh cells in the terminal ileum have been associated with intestinal inflammation in patients with Crohn's disease and may serve as a correlate of disease activity (15). Although all patients with IBD had more activated cTfh cells than NHDs, they had much fewer SARS-CoV-2 spike-specific cTfh cells. Interestingly, despite immune-modulating therapy, spike-specific cTfh cells were sufficient to facilitate the production of detectable spike IgG, RBD IgG, and neutralizing titers.

All patients with IBD had similar frequencies of spike-specific CD4^+^ and CD8^+^ T cells after COVID-19 vaccination, irrespective of immune-modulating therapy. This is encouraging because T-cell-mediated immune responses are needed to prevent severe disease (9,10). Although these findings are limited by the small sample size, they provide further evidence that patients with IBD on immune-modulating therapies are able to mount robust immune responses to the SARS-CoV-2 vaccine.

## CONFLICTS OF INTEREST

**Guarantors of the article:** Brigid S. Boland, MD, and Jennifer M. Dan, MD, PhD.

**Specific author contributions:** Study design: B.S.B. and J.M.D. Recruitment: B.S.B., J.N., H.L., A.E.C., N.H.N., P.S.D., and S.S. Assays: J.M.D., B.G., Z.Z., N.B., Y.K., A.G., and D.W. All authors: final approval.

**Financial support:** This work was supported by NIH K23 DK123406 (B.S.B.), NIH P30 DK120515 (B.S.B.), NIH T15LM011271 (N.H.N.), NIH 75N9301900065 (D.W., A.S.), NIH U19AI142742 (Shane Crotty), and NIH K08 AI135078 (J.M.D.).

**Potential competing interests:** B.S.B. reports consulting fees from Celgene and Takeda and research grants from Prometheus Biosciences and Gilead. A.E.C. reports consulting fees and speaking fees from AbbVie, Bristol Myers Squibb, Janssen, and Takeda. A.S. is a consultant for Gritstone Bio, Flow Pharma, Arcturus Therapeutics, ImmunoScape, CellCarta, Avalia, Moderna, Fortress and Repertoire.

## References

[1] Melmed GY Agarwal N Frenck RW . Immunosuppression impairs response to pneumococcal polysaccharide vaccination in patients with inflammatory bowel disease. Am J Gastroenterol 2010;105:148–54. 19755964 10.1038/ajg.2009.523

[2] Caldera F Hillman L Saha S . Immunogenicity of high dose influenza vaccine for patients with inflammatory bowel disease on anti-TNF monotherapy: A randomized clinical trial. Inflamm Bowel Dis 2019;26:593–602. 10.1093/ibd/izz164 31504526

[3] Pratt PK David N Weber HC . Antibody response to hepatitis B virus vaccine is impaired in patients with inflammatory bowel disease on infliximab therapy. Inflamm Bowel Dis 2018;24:380–6. 29361083 10.1093/ibd/izx001

[4] Harrington JE Hamilton RE Ganley-Leal L . The immunogenicity of the influenza, pneumococcal, and hepatitis B vaccines in patients with inflammatory bowel disease treated with vedolizumab. Crohns Colitis 360 2020;2:otaa082. 10.1093/crocol/otaa082 PMC9802260 36777751

[5] Lee AS Caldera F. Is vedolizumab truly gut selective? It may not affect the immunogenicty of vaccines in patients with inflammatory bowel disease. Crohns Colitis 360 2020;2:otaa086. 10.1093/crocol/otaa086 PMC9802035 36777763

[6] Kennedy NA Lin S Goodhand JR . Infliximab is associated with attenuated immunogenicity to BNT162b2 and ChAdOx1 nCoV-19 SARS-CoV-2 vaccines in patients with IBD. Gut 2021;70:1884–93. 33903149 10.1136/gutjnl-2021-324789

[7] Kappelman MD Weaver KN Boccieri ME . Humoral immune response to mRNA COVID019 vaccines among patients with IBD. Gastroenterology 2021;161:1340–3.e2. 34144046 10.1053/j.gastro.2021.06.016 PMC8321883

[8] Wong S-Y Dixon R Pazos VM . Serological response to mRNA COVID-19 vaccines in IBD patients receiving biological therapies. Gastroenterology 2021;161:715–8.e4. 33887219 10.1053/j.gastro.2021.04.025 PMC8055494

[9] Moderbacher CR Ramirez SI Dan JM . Antigen-specific adaptive immunity to SARS-CoV-2 in acute COVID-19 and associations with age and disease severity. Cell 2020;183:996–1012.e19. 33010815 10.1016/j.cell.2020.09.038 PMC7494270

[10] Dan JM Mateus J Kato Y . Immunological memory to SARS-CoV-2 assessed for up to 8 months after infection. Science 2021;eabf4063. 33408181 10.1126/science.abf4063 PMC7919858

[11] Grifoni A Weiskopf D Ramirez SI . Targets of T cell responses to SARS-CoV-2 coronavirus in humans with COVID-19 disease and unexposed individuals. Cell 2020;181:1489–501.e15. 32473127 10.1016/j.cell.2020.05.015 PMC7237901

[12] Sette A Crotty S. Adaptive immunity to SARS-CoV-2 and COVID-19. Cell 2021;184:861–80. 33497610 10.1016/j.cell.2021.01.007 PMC7803150

[13] Crotty S. T follicular helper cell biology: A decade of discovery and diseases. Immunity 2019;50:1132–48. 31117010 10.1016/j.immuni.2019.04.011 PMC6532429

[14] Song G He W Callaghan S . Cross-reactive serum and memory B-cell responses to spike protein in SARS-CoV-2 and endemic coronavirus infection. Nat Commun 2021;12:2938. 34011939 10.1038/s41467-021-23074-3 PMC8134462

[15] Globig A-M Sommer NP Wild K . Ustekinumab inhibits T follicular helper cell differentiation in patients with Crohn's disease. Cell Mol Gastroenterol Hepatol 2021;11:1–12. 32679193 10.1016/j.jcmgh.2020.07.005 PMC7593584

